# Effect of diode laser pretreatment on dentin thickness and discoloration in deep caries treated with biodentine: a randomized clinical trial

**DOI:** 10.1038/s41598-026-47695-0

**Published:** 2026-05-06

**Authors:** Hanaa Mahmoud Elgamily, Nehal Sharaf, Islam S. Shaker, Amr Shibata, Mohamed B. Abdelhamid

**Affiliations:** 1 Restorative and Dental Materials Department, Oral and Dental Research Institutes, National Research Centre, 33 El Bohouth Street (former El Tahrir Street), Dokki, P.O. 12622, Giza, Egypt; 2 Oral and Maxillofacial Radiology Department, Faculty of Oral and Dental Medicine, Misr International University, Cairo, Egypt; 3 Oral and Dental Research Institutes, National Research Centre, 33 El Bohouth Street (former El Tahrir Street), Dokki, P.O. 12622, Giza, Egypt; 4 Operative Department, Faculty of Oral and Dental Medicine, Misr International University, Cairo, Egypt

**Keywords:** Vital pulp therapy, Biodentine, Diode laser, Cone-beam computed tomography (CBCT), Dentin bridge formation, Deep carious lesions., Diseases, Health care, Medical research

## Abstract

Preserving pulp vitality in deep carious lesions remains a clinical challenge. Indirect pulp capping with bioactive calcium silicate materials such as Biodentine has shown favorable biological outcomes, while diode laser irradiation has been proposed as an adjunct to enhance hemostasis, disinfection, and pulpal healing. However, evidence regarding their combined use is limited. This randomized controlled clinical trial compared the clinical and radiographic outcomes of indirect pulp capping using Biodentine alone and diode laser pre-treatment followed by Biodentine. Patients with deep carious lesions were randomly allocated to either Biodentine alone (Group I) or diode laser pre-treatment plus Biodentine (Group II). Changes in dentin thickness were evaluated using cone-beam computed tomography at baseline, 3 months, and 12 months. Tooth discoloration was assessed spectrophotometrically using CIE L*a*b* parameters, while pulp vitality, pain, and sensitivity were clinically recorded. Data were analyzed using repeated-measures ANOVA (*p* < 0.05). Both groups demonstrated significant increases in dentin thickness over time (*p* < 0.001). Group II exhibited significantly greater dentin formation at 3 and 12 months compared with Group I (*p* < 0.05). No significant differences in tooth discoloration were observed between groups, and all ΔE values remained within clinically acceptable limits. Pulp vitality was preserved in all cases, with significantly lower postoperative pain scores in the diode laser group. Within the limitations of this randomized clinical trial, diode laser pretreatment was associated with a greater increase in radiographically detectable dentin thickness following vital pulp therapy with Biodentine.

## Introduction

Managing deep carious lesions remains a clinical challenge due to the delicate balance between effective caries removal and preservation of pulp vitality. The goal in treating these lesions is to arrest caries progression while maintaining a functional and vital pulp, thereby avoiding the need for endodontic intervention^1^. Vital pulp therapy (VPT), particularly indirect pulp capping, where a biocompatible material is placed over compromised dentin to stimulate reparative dentin formation and protect the pulp^2,3^. The success of this approach is highly dependent on exposure site criteria, pulp condition, bleeding, biocompatibility, insoluble pulp capping material that stimulates odontoblastic activity and enhances reparative dentin formation, and proper cavity sealing with a restoration after application of the capping material to prevent any bacterial leakage and contamination^4^.

Despite advances in biomaterials and minimally invasive treatment approaches, considerable variability still exists in clinical decision-making regarding vital pulp therapy procedures. A recent multinational survey was conducted involving dental practitioners from 16 countries demonstrated substantial differences in clinicians’ preferences for caries removal strategies and pulp capping materials. The study highlighted that treatment decisions are influenced by multiple clinical factors, including the nature of the pulp exposure, patient age, and perceived risk of treatment failure. Importantly, the survey also indicated a growing preference for calcium silicate-based biomaterials over traditional calcium hydroxide formulations in direct pulp capping procedures^5^.

Calcium hydroxide-based materials are among the most extensively studied and widely accepted options for direct pulp capping, often considered the benchmark for evaluating newer alternatives^6^. However, these materials present several limitations, including inadequate adhesion to dentin, susceptibility to dissolution, and limited mechanical strength. Moreover, the reparative dentin observed after their use may result more from a pulp tissue defense response to irritation than from any true bioinductive effect^7,8^. The material’s high alkalinity (pH around 12.5) can lead to surface tissue damage, causing liquefaction necrosis and the development of a necrotic zone at the interface between the pulp and the material^9^.

Mineral trioxide aggregate (MTA) cements—derived from Portland cement and including products such as ProRoot MTA, MTA-Angelus, and Tech Biosealer—are therapeutic calcium silicate-based materials commonly used in endodontic repair procedures. Initially introduced in a grey formulation^10^, MTA has demonstrated the ability to stimulate proliferation and differentiation of human dental pulp cells^11–13^ and exhibits tissue-conductive properties that support the formation of hard tissues, particularly through the development of a dentin bridge over exposed pulp tissue^14,15^. Compared to traditional water-based calcium hydroxide materials, MTA offers superior biocompatibility, inducing less inflammation and necrosis in the pulp tissue^11,14,16^. However, challenges related to handling difficulties and an extended setting time^10,17^ have prompted the development of newer calcium silicate materials^18–20^.

Among contemporary bioactive materials used in vital pulp therapy, Biodentine has gained significant attention due to its favorable biological and physicochemical properties. As a tricalcium silicate-based biomaterial, Biodentine exhibits excellent biocompatibility, bioactivity, and the ability to stimulate mineralized tissue deposition. In addition, its relatively short setting time, improved handling properties, and reduced discoloration potential compared with other calcium silicate materials have contributed to its increasing clinical adoption. Recent reviews of biomaterials used in restorative dentistry and endodontics have emphasized the growing role of calcium silicate-based materials in promoting pulp healing and supporting regenerative responses within the pulp–dentin complex^21^.

Biodentine (Septodont), a tricalcium silicate-based bioactive cement, has emerged as a promising material for vital pulp therapies. Composed of primarily calcium silicate, calcium carbonate, zirconium oxide, and a hydro soluble polymer, contributes to its excellent biocompatibility and bioactivity^22^. Biodentine exhibits a relatively short setting time (about 12 min) and high compressive strength^23^. It releases calcium hydroxide during setting, creating an alkaline environment conducive to antibacterial activity and promoting dentin bridge formation^24^. Biodentine has demonstrated excellent biocompatibility and the ability to stimulate odontoblastic differentiation, contributing to reparative dentinogenesis^25,26^. Several clinical and histological studies have validated its efficacy in managing deep caries without pulpal exposure, reporting favorable outcomes in terms of pulp vitality and dentin bridge thickness, making it a material of choice for indirect pulp capping^27,28^. In addition, it has been claimed that Biodentine causes less coronal discoloration than MTA^29–31^, as zirconium oxide did not result in discoloration, and, importantly, did not influence the hydration of the material^32,33^.However, a review article reported that teeth discoloration using Biodentine is highly expected when material is placed in direct contact with blood during dental procedures. While in the absence of blood, Biodentine causes less teeth color changes than MTA-based materials, but it is still unclear what clinically relevant results could be expected regarding the discoloration frequency and intensity induced by Biodentine^34^.

In recent years, diode lasers, particularly those in the 940 nm wavelength range, have been effectively utilized in direct pulp capping (DPC) procedures, primarily due to their ability to provide excellent hemostasis and sterilization during treatment. These lasers offer benefits such as effective bleeding control, microbial reduction, and photobiomodulation, all of which contribute to favorable healing responses^33,34^. Furthermore, some preclinical and preliminary clinical evidence suggests that laser irradiation might modify the dentin surface, potentially improving the adhesion of restorative materials^35^. While direct stimulation of dentinogenesis by diode lasers is not as widely established as with materials like Biodentine, some studies propose that laser energy could influence cellular activity, potentially contributing to reparative dentin formation^36,37^.

Cone Beam Computed Tomography (CBCT) has emerged as a valuable diagnostic tool in endodontic and restorative dentistry, offering high-resolution, three-dimensional imaging with relatively low radiation exposure compared to conventional CT. In the context of vital pulp therapy, CBCT provides a non-invasive and accurate method to assess dentin bridge formation over time. Unlike two-dimensional radiographs, CBCT enables detailed visualization of the internal tooth structure, including the location, continuity, and thickness of reparative dentin, with enhanced clarity and spatial orientation^38^. This technology allows clinicians and researchers to quantitatively evaluate changes in dentin over follow-up periods, thereby offering a reliable means of monitoring tissue regeneration and treatment outcomes^39^. In this study, CBCT was utilized to compare the increase in dentin thickness between two treatment modalities—Biodentine alone and diode laser pre-treatment followed by Biodentine—at 6 and 12 months, thus serving as an objective and reproducible tool for assessing pulpal healing and hard tissue deposition.

Despite the individual merits of Biodentine and diode lasers in managing deep carious lesions, the combined effect of diode laser pre-treatment followed by Biodentine placement remains largely under-researched. There is a clear need to investigate whether this combined approach can lead to synergistic effects, potentially enhancing reparative dentin formation and improving overall treatment outcomes compared to Biodentine alone. This study aims to address this knowledge gap by rigorously comparing the efficacy of these two treatment modalities.

Therefore, the purpose of this study was to evaluate and compare the radiographic and clinical outcomes of managing deep carious lesions using Biodentine alone versus diode laser pre-treatment followed by Biodentine application. Specifically, this investigation aimed to assess changes in dentin thickness using cone beam computed tomography (CBCT) at 6 and 12 months post-treatment, as well as to monitor tooth discoloration and clinical parameters such as pain, sensitivity, and pulp vitality during follow-up. The null hypothesis of this study was that there would be no significant difference between the two treatment protocols—Biodentine alone and diode laser-assisted Biodentine application—with respect to reparative dentin thickness, tooth discoloration, and clinical outcomes over the observation period.

## Materials and methods

### Study design and ethical approval

This study was designed as a randomized controlled clinical trial with a parallel-group design. The trial was registered in the Clinical Trials Registry (ClinicalTrials.gov) under the identification number #NCT07358442, 22/01/2026. All procedures were conducted in accordance with the ethical principles outlined in the Declaration of Helsinki and followed applicable national and institutional regulations. Ethical approval was obtained from the Medical Research Ethics Committee, National Research Centre, Egypt (Approval No. 03441225). Written informed consent was obtained from all participants prior to enrollment.

### Study population and case selection

Participants were recruited from patients seeking dental care and were included based on the following criteria: Adults aged 18–45 years, in good general health, willing to participate and comply with follow-up visits. Presence of a single deep carious lesion in a restorable permanent posterior molar diagnosed with reversible pulpitis or asymptomatic vital pulp, confirmed by clinical examination, pulp vitality tests, and radiographic assessment. Radiographic evidence of deep caries extending to the inner half of dentin, approaching but not exposing the pulp. Absence of clinical or radiographic signs of irreversible pulpitis or periapical pathology. While patients were excluded if any of the following conditions were present: Ongoing orthodontic treatment, significant periodontal disease affecting the study tooth, or inability to tolerate CBCT imaging. Medically compromised conditions that may affect healing or mineralization (e.g., uncontrolled diabetes). Pregnancy, known allergy to Biodentine or any study materials, or poor patient cooperation. Teeth diagnosed with irreversible pulpitis or necrotic pulp, evidenced by spontaneous pain, prolonged response to vitality testing, or pain on percussion. Teeth with periapical radiolucency, previous endodontic treatment, or extensive restorations. Teeth with severe structural destruction requiring full coronal coverage^1–3^.

### Sample size calculation

Sample size estimation was performed based on data reported by Ahmed et al. (2024)^42^, who evaluated dentin bridge formation following diode laser–assisted pulp capping. Assuming an expected mean difference in dentin thickness of 0.35 mm, a standard deviation of 0.40 mm, a significance level of α = 0.05, and a statistical power of 80%, a minimum of 21 participants per group was required. To compensate for an anticipated 10% dropout rate, a total of 48 patients (24 per group) were recruited to ensure adequate statistical power.

### Randomization and blinding

Participants were randomly assigned to one of two treatment groups using computer-generated random numbers (www.randomizer.org). Group assignments were placed in sequentially numbered, opaque, sealed envelopes to ensure allocation concealment.

**The randomization** sequence and envelope preparation were performed by an independent research coordinator not involved in patient recruitment or treatment. Envelopes were opened only after patient eligibility confirmation and informed consent, immediately before treatment.

For the blinding.

- Patient blinding: Participants were blinded to group allocation. Laser application was performed in a manner that minimized perceptible differences between interventions.
- To ensure patient blinding, the same clinical setup was used in both groups. In the experimental group, the diode laser was activated and applied according to the study parameters. In the control group, the laser handpiece was positioned in the same manner but was not activated (sham irradiation). Because laser application was performed in non-contact mode and produced minimal sensory perception, participants were unable to distinguish between active and sham procedures.
- Operator blinding: Blinding of the operator was not feasible due to the nature of laser application; however, standardized treatment protocols were strictly followed.
- Outcome assessor blinding:
  - CBCT images were anonymized and independently evaluated by two experienced oral and maxillofacial radiologists, both blinded to treatment allocation.
  - Tooth color assessments were performed by an independent blinded examiner at the Medical Research Centre of Excellence, National Research Centre.
- Statistical blinding: The statistician analyzed coded datasets without knowledge of group allocation.

### Intervention protocols

All procedures were performed under local anesthesia and rubber dam isolation, following strict aseptic protocols. Baseline pain scores were recorded using the Visual Analog Scale (VAS) prior to administration of local anesthesia and placement of the rubber dam to avoid interference from anesthetic effects.

#### Group 1: Biodentine Group (Control)

**Caries removal**

Selective caries removal was performed to firm or leathery dentin, preserving affected dentin adjacent to the pulp in accordance with contemporary minimally invasive caries management principles^43^:

- Undermined enamel was removed using a #245 high-speed carbide bur.
- Soft infected dentin was removed using a spoon excavator.
- Firm, leathery dentin adjacent to the pulp was carefully refined using a slow-speed sterile round bur.
- In cases of pinpoint mechanical pulp exposure, hemostasis was achieved using gentle pressure with moist and dry cotton pellets. However, No cases of mechanical pulp exposure occurred during the selective caries removal procedures in either the laser-assisted group or the control group.

**Pulp protection**

Biodentine (Septodont, France) was mixed and applied according to the manufacturer’s instructions and placed directly over the exposed or near-exposed dentin without condensation. Initial setting time was approximately 12 min.

**Restoration**

After Biodentine setting, cavities were restored with resin-modified glass ionomer cement (Fuji II LC, GC Corporation, Japan).

#### Group 2: Diode Laser + Biodentine Group (Experimental)

Caries removal and exposure management were identical to Group 1.

Caries removal

Immediately after exposure control, a 940 nm diode laser (EPIC X™, BIOLASE, USA) was applied using the following parameters^42^:

- Wavelength: 940 nm.
- Output power: 0.1 W.
- Mode: Continuous wave.
- Duration: 10 s.
- Fiber tip: A non-initiated 400 μm fiber tip was used to deliver low-level laser irradiation in non-contact mode.
- Technique: Non-contact sweeping motion at ~ 1 mm distance from pulp tissue.

Laser application parameters were selected based on reported photobiomodulatory and antibacterial effects on pulp tissue^36,38,39^.

All safety precautions were followed, including laser protective eyewear, warning signage, and device calibration.

Prior to each clinical session, the diode laser device was calibrated according to the manufacturer’s instructions using a laser power meter, to ensure consistent power output.

**Pulp protection and restoration**

Biodentine placement and final restoration were performed identically to the control group.

### Outcome measures and data collection

Data were collected at baseline (T0), 6 months (T1), and 12 months (T2) using standardized protocols.

### Clinical assessment

- Pulp vitality testing (cold test and EPT).
- Pain and sensitivity assessment using the Visual Analog Scale (VAS).
- Percussion, palpation, and mobility testing.

### CBCT assessment of dentin thickness

CBCT imaging was performed using Cranex 3D (Soredex, Finland) with the following parameters:

- Field of view: 5 × 5 cm.
- Tube current: 8 mA.
- Tube voltage: 90 kVp.
- Voxel size: 0.085 mm.

Images were analyzed using OnDemand3D software, measuring the linear distance from the pulpal floor of the cavity to the roof of the pulp chamber on properly oriented sagittal, coronal, and cross-sectional views^40,41^. Measurements were repeated three times, and mean values were recorded.

To assess measurement reliability, dentin thickness measurements were independently performed by two calibrated radiologists and repeated after a two-week interval under identical conditions. Intra- and inter-examiner reproducibility were evaluated using the intraclass correlation coefficient (ICC), which demonstrated high agreement.

### Tooth color assessment

Tooth color was evaluated using:

- Standardized digital photography.
- VITA Easyshade^®^ Advance spectrophotometer.

Color parameters (L*, a*, b*, h, C*) were recorded according to the CIE Lab system*^28,29^. Each measurement was repeated three times, and the average was calculated. Total color change (ΔE) was calculated using the formula:

$${\mathrm{\boldsymbol{\Delta}}}E=\sqrt {\left( {{\mathrm{\boldsymbol{\Delta}}}L{)^2}+{{({\mathrm{\boldsymbol{\Delta}}}a)}^2}+{{({\mathrm{\boldsymbol{\Delta}}}b)}^2}} \right.}$$

### Examiner calibration and reliability

All examiners underwent calibration sessions prior to data collection. Intra- and inter-examiner reliability for CBCT and color measurements were assessed using intraclass correlation coefficients (ICCs).

### Statistical analysis

Statistical analysis was performed using IBM SPSS Statistics v26.0 (IBM Corp., USA). Continuous data were expressed as mean ± standard deviation, while categorical data were expressed as frequencies and percentages. Normality of the data distribution was assessed using the Shapiro–Wilk test within SPSS prior to performing parametric statistical analysis.

For dentin thickness, a repeated measures analysis of variance (ANOVA) was used to evaluate changes over time within subjects as well as differences between the two treatment groups. When significant effects were detected, post hoc pairwise comparisons were performed with appropriate adjustment. Tooth color changes (ΔE) were analyzed using parametric or non-parametric tests as appropriate. VAS pain scores were analyzed to assess changes over time within each group and differences between groups at each follow-up interval using parametric tests, as appropriate. Categorical outcomes were analyzed using Chi-square or Fisher’s exact tests. Statistical significance was set at *p* < 0.05.

## Results

### Study population and follow-up

**Fig. 1:**
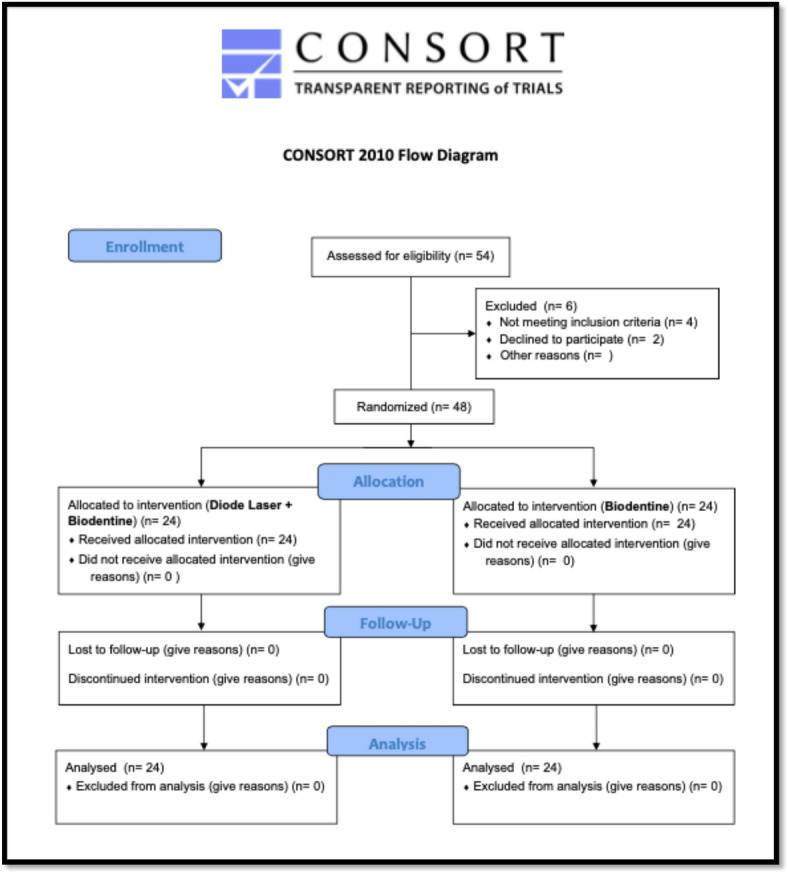
CONSORT flow diagram.

A total of 48 patients with deep carious lesions were enrolled and randomly allocated into two equal groups (*n* = 24 each): Group I (Biodentine alone) and Group II (diode laser pre-treatment followed by Biodentine). All patients completed the 6-months follow-up with no dropouts. No statistically significant differences were found between groups regarding age, sex, or baseline clinical parameters (*p* > 0.05) (Fig. 1).

### Radiographic evaluation (CBCT – dentin thickness)

**Fig. 2:**
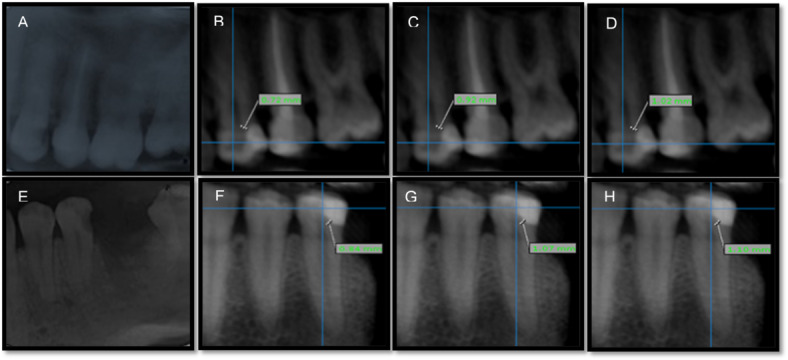
Preoperative Periapical Radiograph of upper 2nd premolar with carious lesion involving 2/3 of dentin & CBCT cross-sectional images showing increased residual dentin thickness at baseline, 3 months, and 6 months for Biodentine group in A, B, C& D respectively. Preoperative Periapical Radiograph of lower 2nd premolar with carious lesion involving 2/3 of dentin & CBCT cross-sectional images showing increased residual dentin thickness at baseline, 3 months, and 6 months for Diode laser group in E, F, G & H respectively.

CBCT analysis revealed a significant increase in residual dentin thickness over time in both groups (*p* < 0.001) (Fig. 2).

- **Group I (Biodentine alone)** showed a gradual and significant increase in dentin thickness at 3 and 6 months compared to baseline.
- **Group II (Diode laser + Biodentine)** demonstrated a significantly greater increase in dentin thickness at both follow-up intervals compared to Group I (*p* < 0.05).

Repeated measures ANOVA demonstrated a significant effect of time and a significant interaction between time and treatment group, indicating that the pattern of dentin thickness change differed between groups.

### Radiographic outcomes (CBCT – residual dentin thickness)

Both groups showed a significant increase in residual dentin thickness over time (*p* < 0.001). Group II demonstrated significantly greater dentin formation at both 3 and 6 months compared to Group I (Tables 1, 2, 3, 4, and 5).

**Table 1:** Changes in residual dentin thickness (mm) assessed by CBCT.

Time interval	Group I (Biodentine) Mean ± SD	Group II (Laser + Biodentine) Mean ± SD	*p*-value
Baseline	0.62 ± 0.11	0.61 ± 0.10	0.78
3 months	0.91 ± 0.13	1.12 ± 0.14	**0.002**
6 months	1.18 ± 0.15	1.45 ± 0.17	**< 0.001**

Significant values are in [bold].

**Within-group comparison**: *p* < 0.001 for both groups. **Between-group comparison**: Independent t-test.

#### Measurement reliability

Intra- and inter-examiner reliability analysis demonstrated high agreement for dentin thickness measurements, as assessed by intraclass correlation coefficients (ICCs).

#### Tooth discoloration assessment

Color changes assessed by standardized digital photography, spectrophotometry (VITA Easyshade^®^), and CIE L*a*b* analysis revealed:

- Minimal color change in both groups over the 6-month follow-up period.
- No statistically significant differences between groups in ΔE values at any evaluation interval (*p* > 0.05).

All recorded ΔE values remained within clinically acceptable limits, indicating that diode laser pre-treatment did not adversely affect esthetic outcomes.

#### Tooth discoloration analysis

Color measurements revealed minimal changes over time in both groups. No statistically significant differences were observed between groups at any evaluation interval.

**Table 2:** CIE Lab Color Changes (ΔE Values) *.

Time Interval	Group I Mean ± SD	Group II Mean ± SD	*p*-value
3 months	2.05 ± 0.63	2.12 ± 0.58	0.67
6 months	2.38 ± 0.71	2.41 ± 0.69	0.81

All ΔE values were **below the clinically perceptible threshold (ΔE < 3.3)**.

### Clinical outcomes

#### Pulp vitality

All teeth in both groups remained vital throughout the study period, with no statistically significant difference between groups at any follow-up interval (*p* > 0.05).

**Table 3:** Pulp vitality outcomes.

Follow-up	Group I (Vital %)	Group II (Vital %)	*p*-value
3 months	100%	100%	1.00
6 months	100%	100%	1.00

#### Postoperative sensitivity and pain

VAS scores showed a significant reduction in postoperative sensitivity and pain over time in both groups (*p* < 0.001).

- Group II exhibited significantly lower VAS scores at both 3 and 6 months compared to Group I (*p* < 0.05), indicating improved patient comfort following diode laser application.

VAS scores decreased significantly in both groups over time. Group II exhibited significantly lower pain scores at both follow-up intervals.

**Table 4:** VAS pain scores.

Time interval	Group I Mean ± SD	Group II Mean ± SD	*p*-value
Baseline	5.8 ± 1.1	5.6 ± 1.2	0.61
3 months	2.3 ± 0.9	1.5 ± 0.7	**0.01**
6 months	1.6 ± 0.6	0.9 ± 0.5	**0.003**

Significant values are in [bold].

#### Clinical success rate

Clinical success—defined as absence of spontaneous pain, normal response to vitality tests, and lack of radiographic pathology—was high in both groups. However, Group II demonstrated a slightly higher overall success rate, although this difference was not statistically significant (*p* > 0.05).

**Table 5:** Clinical success rate at 6 months.

Outcome	Group I	Group II	*p*-value
Successful cases	22 (92%)	23 (95%)	0.55
Failed cases	2 (8%)	1 (5%)	

### Adverse events

Three cases were classified as clinical failures during the follow-up period. Two failures occurred in the Biodentine group and one in the diode laser group. These teeth presented with persistent pain and abnormal pulp responses suggestive of irreversible pulpitis. Endodontic treatment was subsequently performed. No cases developed periapical pathology during the observation period.

## Discussion

The present randomized controlled clinical trial investigated the effect of diode laser pre-treatment prior to Biodentine application on reparative dentin formation, tooth discoloration, and clinical outcomes in deep carious lesions. Preserving pulp vitality in such cases is a central goal of contemporary minimally invasive dentistry, where selective caries removal and biologically driven materials are preferred over aggressive excavation strategies. This approach aligns with current recommendations emphasizing pulp preservation and controlled healing responses in deep caries management^1,2^^3^.

Although all teeth were clinically diagnosed with reversible pulpitis, it is recognized that pulp inflammation represents a biological continuum that cannot be precisely determined using clinical diagnostic methods alone. Variations in the pre-existing inflammatory status of the pulp may therefore influence treatment outcomes and should be considered when interpreting the findings^43^.

The laser irradiation parameters used in the present study (0.1 W for 10 s) were selected based on previously reported protocols investigating photobiomodulation effects in dental tissues. The total delivered energy was approximately 1 J per tooth. Although some studies have suggested that photobiomodulation effects may be more pronounced at higher energy doses, lower energy levels have also been reported to stimulate cellular activity while minimizing the potential risk of thermal damage to pulpal tissues. Given the delicate nature of the pulp–dentin complex, conservative irradiation parameters were selected in the present study to ensure biological safety while still achieving potential photobiomodulatory effects^42^.

Radiographic analysis demonstrated a significant increase in dentin thickness over time in both groups, confirming the bioactive potential of Biodentine in stimulating reparative dentinogenesis. Biodentine is a tricalcium silicate–based material known to release calcium ions and create an alkaline environment that promotes pulp cell differentiation and mineralized tissue formation. These findings are consistent with previous clinical and tomographic studies reporting favorable dentin bridge formation and pulp healing following indirect or direct pulp capping with Biodentine^22,25,28^.

Notably, teeth treated with diode laser pre-treatment followed by Biodentine exhibited significantly greater dentin thickness at both 3- and 6-month follow-ups compared with Biodentine alone. The beneficial effects observed in the diode laser group may be partly explained by photobiomodulation mechanisms. Low-level laser irradiation has been shown to enhance mitochondrial activity, increase ATP production, and stimulate proliferation and differentiation of dental pulp stem cells. Experimental studies have demonstrated that PBM can upregulate odontogenic markers and promote mineralized tissue formation. These cellular effects provide a plausible biological explanation for the enhanced mineralized tissue deposition observed in the present clinical trial^44^. Such biological stimulation likely synergizes with the bioactive properties of calcium silicate materials, accelerating reparative dentin formation^36,38,39^.

In addition to photobiomodulation effects, diode laser irradiation may also contribute to improved outcomes through its antimicrobial activity. Laser energy has been shown to reduce bacterial load within dentinal tubules and the surrounding dentin substrate, thereby decreasing microbial contamination at the pulp exposure site. This antimicrobial effect may help create a more favorable environment for pulp healing and mineralized tissue deposition. Experimental and clinical evidence has suggested that laser-assisted disinfection may enhance the success of vital pulp therapy procedures by reducing residual bacterial contamination and promoting a more stable biological environment for tissue repair^45^.

The use of CBCT in this study enabled accurate three-dimensional assessment of dentin thickness changes over time, overcoming limitations associated with conventional two-dimensional radiography. CBCT has been increasingly advocated for endodontic and pulp therapy research due to its superior spatial resolution and reproducibility in measuring hard tissue changes. The standardized imaging protocol and blinded assessment further enhanced measurement reliability and internal validity^40,41^.

Although an increase in dentin thickness was observed radiographically in the present study, it should be emphasized that cone-beam computed tomography cannot differentiate between various types of mineralized tissues. The newly formed tissue may represent tubular dentin, osteodentin, or other calcified structures. Therefore, the radiographic findings should be interpreted as mineralized tissue deposition rather than definitive reparative dentin formation. Histological investigations remain the gold standard for confirming the structural characteristics of newly formed tissue. Previous studies have demonstrated through histological analysis that the biological response of the pulp following vital pulp therapy can involve different patterns of mineralized tissue formation depending on the treatment conditions^43^.

It should be emphasized that the radiographic increase in dentin thickness observed in this study does not necessarily represent true tubular dentin formation, as CBCT imaging cannot distinguish between different types of mineralized tissues. Histological studies remain necessary to confirm the exact structural nature of the newly formed tissue.

Tooth discoloration is a recognized concern associated with calcium silicate–based materials, particularly in esthetically sensitive areas. In the present study, both groups demonstrated minimal color changes over the observation period, with ΔE values remaining below clinically perceptible thresholds. These findings are consistent with previous reports indicating that Biodentine exhibits superior color stability compared with MTA, largely due to the absence of bismuth oxide and the presence of zirconium oxide as a radiopacifier^31,33,34^.

Tooth discoloration associated with calcium silicate-based materials is recognized as a multifactorial phenomenon influenced by several variables, including material composition, blood contamination, environmental exposure, and restoration thickness. Systematic reviews have reported that although Biodentine demonstrates improved color stability compared with mineral trioxide aggregate, discoloration may still occur under certain clinical conditions. The relatively short follow-up period in the present study represents a limitation, and longer-term investigations are required to fully assess the esthetic stability of these materials^46,47^.

Another important factor influencing the success of vital pulp therapy is the quality of the coronal seal. A resin-modified glass ionomer cement was selected as the definitive restoration due to its favorable sealing ability, chemical adhesion to dentin, and fluoride release. Previous studies have shown that the interface between calcium silicate-based materials and restorative materials plays a critical role in preventing bacterial microleakage. Although composite resins may offer superior mechanical properties, RMGIC provides reliable bonding to dentin and is commonly used in vital pulp therapy procedures^48–50^.

Importantly, diode laser application did not increase discoloration risk, suggesting that laser irradiation does not adversely affect material color stability or enamel optical properties. This observation is in agreement with spectrophotometric studies showing that discoloration associated with calcium silicate materials is more strongly influenced by blood contamination and material composition rather than adjunctive laser use^28,31,32^.

Clinically, all treated teeth maintained pulp vitality throughout the follow-up period, indicating a high success rate for both treatment protocols. Reduced postoperative pain scores observed in the diode laser group may be explained by the laser’s ability to reduce bacterial load, seal dentinal tubules, and modulate inflammatory mediators, resulting in decreased pulpal irritation. Similar analgesic and anti-inflammatory effects of low-level laser therapy in vital pulp procedures have been previously reported^33–35^.

Despite the promising findings, several limitations of the present study should be acknowledged. First, although VAS scores were recorded longitudinally, subject-level responder analysis was not performed. Such analysis could provide deeper insight into individual patient pain trajectories and variability in treatment response. Future studies should incorporate longitudinal subject-level tracking to enable more comprehensive evaluation of patient-reported outcomes.

Second, measurement reproducibility was assessed using intraclass correlation coefficients (ICCs), which demonstrated high agreement between repeated measurements. However, numerical ICC values were not reported, which may limit the quantitative assessment of reproducibility. In addition, further analytical approaches, such as regression analysis of repeated measurements, were not performed. While ICC remains a widely accepted method for assessing reliability, incorporating complementary statistical techniques could further strengthen the robustness of measurement validation in future investigations.

Furthermore, the follow-up period was limited to six months, which may not fully reflect the long-term clinical and radiographic outcomes of vital pulp therapy. Longer follow-up periods are required to evaluate the durability of treatment outcomes and the long-term stability of restorative materials.

In addition, although cone-beam computed tomography (CBCT) allowed three-dimensional evaluation of changes in dentin thickness, this imaging modality cannot differentiate between various types of mineralized tissues. Therefore, the radiographic increase in dentin thickness observed in this study cannot be interpreted as definitive evidence of true tubular dentin formation, as histological confirmation would be required for such determination.

Moreover, although all teeth were clinically diagnosed with reversible pulpitis, the true inflammatory status of the pulp cannot be precisely determined using clinical diagnostic tests alone, and variations in baseline pulpal inflammation may have influenced treatment outcomes. Finally, although the sample size was adequate for detecting differences in the primary outcome, remains relatively modest. Future studies with larger sample sizes, longer follow-up periods, and integration of biological or histological assessments are recommended to further validate the findings of the present investigation^4,6,14^.

Within these limitations, the findings of the present study suggest that diode laser pre-treatment may enhance radiographically detectable dentin thickness without compromising esthetic outcomes or pulp vitality. This combined approach represents a promising adjunct in the conservative management of deep carious lesions and aligns with current trends toward minimally invasive and biologically oriented dentistry.

## Conclusion

Within the limitations of this randomized controlled clinical trial, the adjunctive use of diode laser irradiation prior to vital pulp therapy with Biodentine was associated with a greater increase in radiographically detectable dentin thickness compared with the conventional protocol. Both treatment approaches demonstrated favorable clinical outcomes and maintained pulp vitality during the six-month follow-up period.

The observed radiographic increase in dentin thickness may indicate enhanced mineralized tissue deposition; however, the exact structural nature of the newly formed tissue cannot be determined using CBCT imaging alone. Therefore, these findings should be interpreted with caution.

The potential biological effects of diode laser irradiation, including photobiomodulation and antimicrobial activity, may contribute to the favorable healing response observed in the laser-assisted group. Nevertheless, further investigations with larger sample sizes, longer follow-up periods, and histological evaluation are required to better understand the biological mechanisms underlying these clinical outcomes.

Diode laser pretreatment may represent a promising adjunctive approach for enhancing radiographic outcomes in vital pulp therapy for teeth with deep carious lesions.

## Data Availability

The datasets used and/or analysed during the current study available from the corresponding author on reasonable request.
